# Comparison of self-reported sleep sufficiency and accelerometer-measured sleep duration in relation to mental health, physical health, and life satisfaction

**DOI:** 10.3389/frsle.2025.1661250

**Published:** 2025-11-26

**Authors:** Hannah Ahrensberg, Anne Illemann Christensen, Susan Andersen, Christina Bjørk Petersen

**Affiliations:** 1 National Institute of Public Health, University of Southern Denmark, Copenhagen, Denmark; 2 Center for Clinical Research and Prevention Copenhagen University Hospital – Bispebjerg and Frederiksberg, Capital Region of Denmark, Copenhagen, Denmark

**Keywords:** cross-sectional study, sleep sufficiency, sleep duration, accelerometer, SF-12, Cantril ladder

## Abstract

**Introduction:**

Sleep is crucial for health and wellbeing, but different dimensions of sleep may affect health differently. This cross-sectional study explores the associations of self-reported sleep sufficiency and accelerometer-measured sleep duration with mental health, physical health, and life satisfaction.

**Materials and methods:**

Data from 1,022 individuals (age ≥16 years) from the Danish Health and Morbidity Survey in 2023 were used. Mental and physical health were assessed using the SF-12 questionnaire, and life satisfaction with the Cantril Ladder scale. Multiple adjusted linear regression models were used to examine associations separately and in four combined categories: (1) low sufficiency, <7/>9 h (*n* = 106), (2) low sufficiency, 7–9 h (*n* = 89), (3) high sufficiency, <7/>9 h (*n* = 271), and (4) high sufficiency, 7–9 h (*n* = 556).

**Results:**

Deviations from recommended sleep durations (<7 or >9 h) and low sleep sufficiency were associated with poorer mental health, physical health and life satisfaction, most strongly for mental health and life satisfaction. Specifically, individuals sleeping 7–9 h with low perceived sleep sufficiency had mental health scores of 10.9 points (95% CI: −13.2; −8.6) lower than those sleeping 7–9 h and reporting high sleep sufficiency. Similarly, those sleeping <7/>9 h and reporting low sleep sufficiency had mental health scores 8.5 points (95% CI: −10.8; −6.3) lower.

**Conclusion:**

Regardless of sleep duration, low sleep sufficiency was consistently associated with poorer health outcomes, suggesting that self-reported sleep sufficiency may be more correlated to health than accelerometer-measured sleep duration alone. These findings underscore the need to integrate multiple sleep dimensions and measurement strategies into public health surveillance.

## Introduction

Poor sleep is common in many western countries (Liu et al., 2016; Kronholm et al., 2016; Chaput et al., 2017; Chattu et al., 2018), although sleep is essential for health and wellbeing (Irwin, 2015; Goel et al., 2009; Spytska, 2024). This poses a significant health and economic burden and represents a major public health concern (Chaput et al., 2020). In Denmark, insufficient sleep is also prevalent, with a notable increase from 10% in 2013 to 16% in 2023 (Ahrensberg et al., 2025).

Sleep is a multidimensional concept, with no clear definition of what constitutes “healthy” sleep. Buysse (2014) proposed a widely used framework defining healthy sleep across several dimensions, including sleep duration, satisfaction, continuity or efficiency, timing, and sustained alertness during waking hours (Buysse, 2014). Measuring all these dimensions simultaneously is challenging, and studies often focus on one or a few dimensions.

International studies have traditionally focused on sleep duration (Matricciani et al., 2018), often using study-specific definitions of short and long sleep, and have shown associations with negative health outcomes such as all-cause mortality, cardiovascular diseases, and depression (Alvarez and Ayas, 2004; Cappuccio et al., 2011, 2010; Damgaard et al., 2024). More recently, attention has shifted toward sleep quality as an important dimension of sleep health (Pilcher et al., 1997; Liu et al., 2013; Kohyama, 2021). Sleep quality captures multiple aspects of healthy sleep, including insomnia symptoms, perceived restfulness, daytime sleepiness, and perceived sleep satisfaction (Kohyama, 2021). Studies focusing on sleep quality, often through measures of insomnia, also report adverse health effects (Damgaard et al., 2024; Sofi et al., 2014; Li et al., 2014; Clement-Carbonell et al., 2021; Scott et al., 2021). However, the impact of sleep duration and sleep quality on health may differ.

Understanding how both sleep duration and sleep quality are related to mental health and physical health is essential for developing targeted public health strategies. Only few studies have examined the joint effect of sleep quality and sleep duration on health (Lallukka et al., 2018; Matsui et al., 2021; Seow et al., 2020). These studies suggest that poor sleep quality is linked to poorer mental health and wellbeing, physical health, and social functioning regardless of sleep duration (Lallukka et al., 2018; Matsui et al., 2021; Seow et al., 2020). However, these studies categorize sleep duration into <6 h, 6–8 h, >8 h, rather than using cut-points based on the recommended levels defined by the National Sleep Foundation's recommendations for adults (7–9 h) (Hirshkowitz et al., 2015). Furthermore, these studies rely on retrospective self-reports of sleep duration, which may lack accuracy and known to be overestimated (Lauderdale et al., 2008). More accurate measures, such as device-based measures, are therefore preferable (Grandner, 2019).

Furthermore, it is highly relevant to include multiple dimensions of wellbeing to fully capture a nuanced understanding of how sleep is associated with overall wellbeing (Frijters and Krekel, 2021; Rasmussen et al., 2025). Previous studies on the combined effects of sleep quality and sleep duration have primarily focused on wellbeing in terms of social and emotional functioning (Lallukka et al., 2018; Matsui et al., 2021). These measures capture psychological functioning at a given point in time but may fluctuate over short periods. Life satisfaction, as a single-measure, is criticized for being less nuanced than multidimensional measures, as it reflects a more overall assessment rather than specific aspects of mental wellbeing (Ruggeri et al., 2020). However, since different wellbeing measures are sensitive to different health-related factors (Rasmussen et al., 2025), life satisfaction remains a valuable complement, especially because it does not refer to a specific time frame (Frijters and Krekel, 2021), is simple, and is relatively stable over time (Lucas et al., 2018). To fully understand wellbeing, it is essential to assess both immediate emotional and psychological dimensions (mental health) and overall life evaluation (life satisfaction), ensuring a more comprehensive approach.

This study aims to explore both the individual and joint associations of sleep sufficiency and sleep duration with mental health, physical health, and life satisfaction. Sleep sufficiency is derived from a self-reported single-item that reflects perceived sleep quality, whereas sleep duration is measured by accelerometer and categorized according to the National Sleep Foundation's recommendations for adults: <7 h, 7–9 h, >9 h. Based on the previous literature, we hypothesize that both low sleep sufficiency and non-recommended sleep duration (shorter or longer than 7–9 h) are associated with poorer mental health, physical health, and lower life satisfaction. The goal is to gain a deeper understanding of the link between sleep and health in order to inform population-based surveillance and guide public health interventions.

## Materials and methods

### Study design and participants

Data were derived from the Danish Health and Morbidity Survey (DHMS) conducted in 2023. The study was approved by the Research and Innovation Organization of the University of Southern Denmark (ID 11.702). DHMS are nationally representative cross-sectional health surveys of the general adult population (≥16 years of age) in Denmark. Individuals were invited to complete the survey either by paper questionnaire or through a web-based survey. The survey sample of 25,000 individuals were randomly drawn from the Danish Civil Registration System where all citizens with official residence in Denmark are registered by a unique personal registration number (Pedersen, 2011).

**Figure 1:**
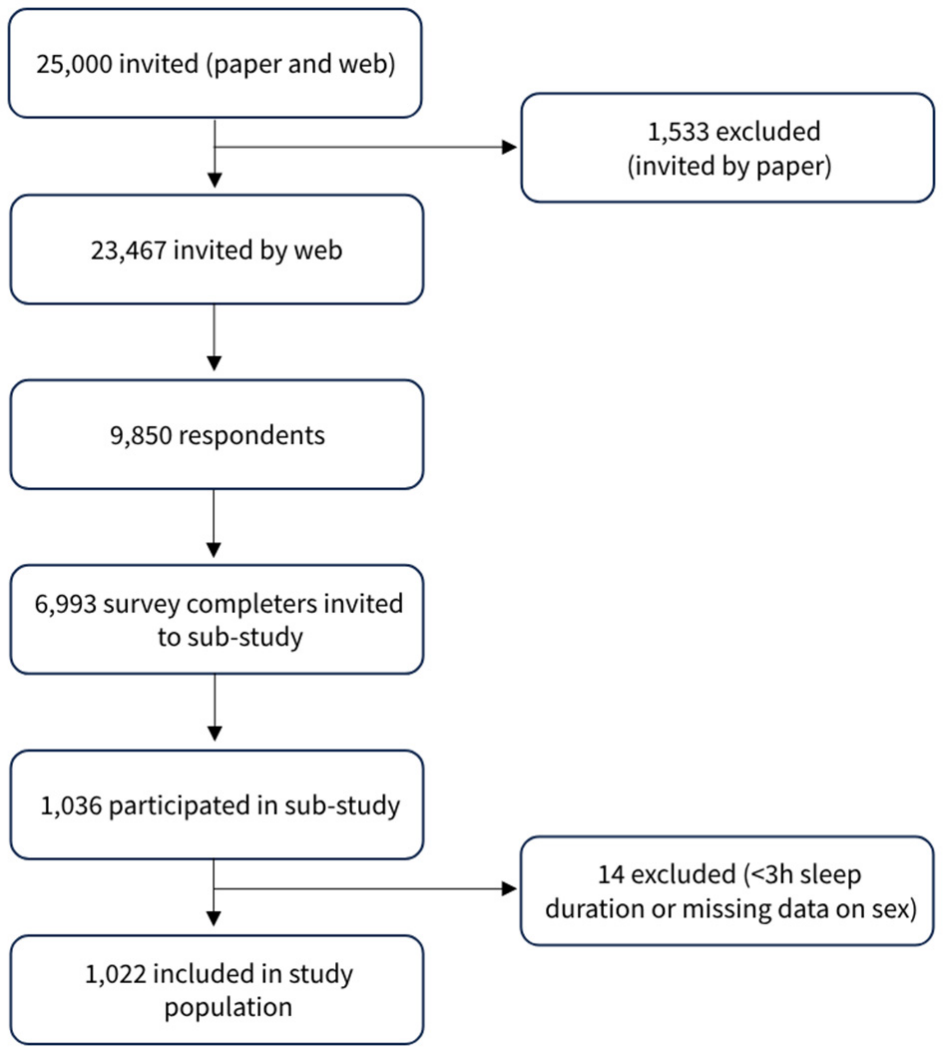
Flowchart of study participants in the Danish Health and Morbidity Survey in 2023.

In the 2023 survey, a nested study was carried out, where a subset of participants in the regular DHMS web-survey also took part in an accelerometer study as a supplement to questionnaire responses on physical activity, sedentary behavior, and sleep. Of the 9,850 individuals who responded to the web-survey, all 6,993 who completed the web-survey were invited to participate in the sub-study (Figure 1). In total, 1,036 participated and wore an accelerometer with at least three valid measurement days (14.8% of those invited). A detailed description of the data collection has been described elsewhere (Jezek et al., 2024). In the present study, participants with an average sleep duration of less than 3 h (*n* = 13) and those with missing data on sex (*n* = 1) were excluded, leaving a total of 1,022 individuals.

### Exposure

#### Sleep sufficiency

Sleep sufficiency was assessed using a single-item question: “Do you think you get enough sleep to feel well-rested?” with the following response options: (1) “Yes, usually,” (2) “Yes, but not often enough” and (3) “No, never (almost never).” Respondents who answered (1) were classified as having high sleep sufficiency, those who answered (2) as having moderate sleep sufficiency, and those who answered (3) as having low sleep sufficiency.

#### Sleep duration

Participants were instructed to wear a SENS motion ^®^ accelerometer on the front of the mid-right thigh for seven consecutive days and to record their sleep periods daily in a sleep diary using the Motus-app (Crowley et al., 2023). Data were collected and processed using the Motus system (version 1.2.2). Although wrist-worn devices are most commonly used for sleep measurement (Ancoli-Israel et al., 2003; Martin and Hakim, 2011), thigh-worn accelerometers are increasingly used as they better distinguish between postures, intensities, and movement behaviors (Stamatakis et al., 2020; Stevens et al., 2020; Johansson et al., 2023). Sleep duration was derived from accelerometer-detected lying periods within the self-reported sleep window recorded in diaries. Only the primary sleep period was included (naps excluded) and any periods of light, moderate or vigorous activity during this window were subtracted from total sleep duration. A valid measurement day was defined as at least 10 h of wake time, and participants with fewer than three valid days were excluded. This threshold was chosen pragmatically to balance data completeness and participant burden to ensure data quality. Average daily sleep duration was calculated as the total sleep time across valid measurement days divided by the number of days. Based on the National Sleep Foundation's recommendations for adults (18–64 years) (Hirshkowitz et al., 2015), sleep duration was categorized into <7 h, 7–9 h and >9 h.

### Outcome

#### Mental health and physical health

Mental health and physical health were assessed using the SF-12 questionnaire (12-Item Short Form Survey), which consists of 12 questions designed to measure the respondent's health status over the past 4 weeks in relation to limitations in mental and physical health (Gandek et al., 1998; Ware et al., 1996). A total score ranging from 0 to 100 was calculated for both the mental health component and the physical health component, with higher scores indicating better health status. All 12 questions contributed to the summary scoring of both components, but the weighting differed depending on the specific component (Christensen et al., 2010). SF-12 has been validated and demonstrated reliability in various populations and settings (Gandek et al., 1998; Ware et al., 1996; Kontodimopoulos et al., 2007; Christensen et al., 2013).

#### Life satisfaction

Life satisfaction was measured using the “Cantril Ladder of Life Scale,” where individuals rated their life satisfaction on a scale from 0 to 10, with 0 representing the worst possible life and 10 the best (Cantril, 1965). The Cantril ladder is a validated and widely accepted tool for measuring life satisfaction (OECD, 2013).

### Covariates

Information on sex and age was obtained from official registers, while information on years of education and chronic disease was collected through survey data. Years of education was categorized into <12 years (primary and upper secondary or vocational education), 13–14 years and ≥15 years of education (higher academic education). Chronic disease was assessed by asking participants: “Do you have any long-standing disease, disorder or illness, long-standing effects of injury, any functional impairment or any other long-standing health problem (six months or more)?” with response options of “Yes” and “No.”

### Statistical analysis

**Figure 2:**
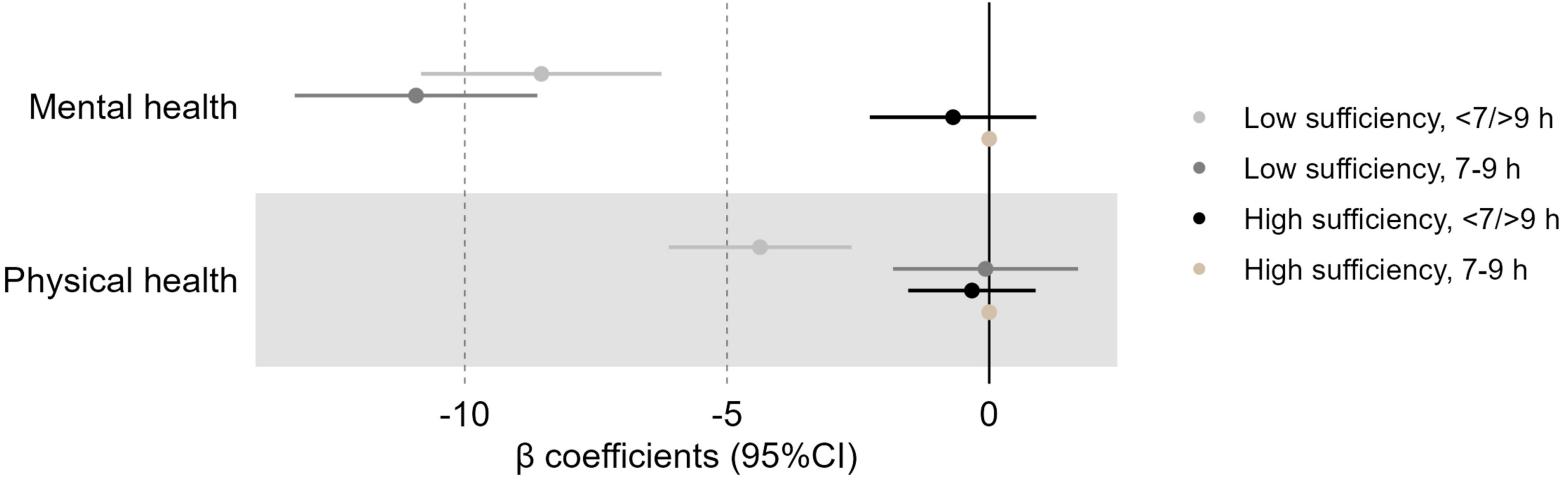
Associations (β coefficients, 95% CI) of sleep duration and sleep sufficiency with mental health and physical health. Adjusted for sex, age, chronic disease, and years of education (weighted data). β, standardized regression coefficient; CI, Confidence Interval. Negative β coefficients indicate lower score of health outcomes.

**Figure 3:**
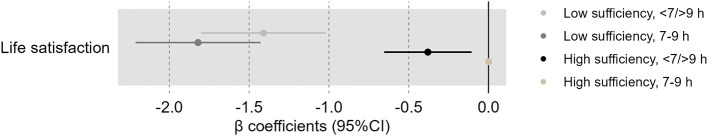
Associations (β coefficients, 95% CI) of sleep duration and sleep sufficiency with life satisfaction. Adjusted for sex, age, chronic disease, and years of education (weighted data). β, standardized regression coefficient; CI, Confidence Interval. Negative β coefficients indicate lower life satisfaction scores.

Descriptive statistics were used for two purposes. First, characteristics of the study population are presented as percentages (Table 1). Second, descriptive associations between exposure and health outcomes are summarized as median values (Table 2). Subsequently, analytical associations were evaluated using multiple adjusted linear regression models. Analyses were conducted separately for sleep sufficiency (low, moderate, high) and sleep duration (<7 h, 7–9 h, >9 h) (Table 3), as well as jointly (Figures 2, 3) by combining sleep sufficiency and sleep duration into four groups: (1) low sufficiency, <7/>9 h, (2) low sufficiency, 7–9 h, (3) high sufficiency, <7/>9 h, and (4) high sufficiency, 7–9 h, with category (4) as the reference group. Moderate and high sleep sufficiency were merged into a single category, labeled high sleep sufficiency, as both reflect generally sufficient sleep, while <7 h and >9 h were combined despite representing distinct sleep phenotypes. This was done to ensure adequate sample sizes in each group for stable estimates, particularly due to the small number of participants sleeping >9 h (*n* = 40) (cf. Supplementary Table S1 for distribution details).

**Table 1:** Characteristics of the study population (*n* = 1,022).

**Characteristic**	***n***	**%**
**Sex**		
Men	374	36.6
Women	648	63.4
**Age**		
16–24 years	62	6.1
25–44 years	253	24.8
45–64 years	458	44.8
65–74 years	194	19.0
≥75 years	55	5.4
**Education**		
<12 years	150	14.7
13–14 years	291	28.5
≥15 years	561	54.9
**Chronic disease**		
Yes	432	42.3
No	590	57.7

Percentages in the table do not sum to 100% due to missing data.

**Table 2:** Median (IQR) of mental health score, physical health score, and life satisfaction score by sleep sufficiency and duration (*n* = 1,022 (weighted data).

**Sleep variable**	***n* (%)**	**Mental health Median (IQR)**	**Physical health Median (IQR)**	**Life satisfaction Median (IQR)**
		49.6 (16.5)	53.5 (10.7)	8.0 (2.0)
**Sleep sufficiency**				
Low	195 (19.1)	37.3 (18.7)	48.4 (18.1)	5.9 (4.0)
Moderate	349 (34.2)	43.9 (15.4)	50.8 (11.0)	7.1 (2.0)
High	478 (46.8)	51.1 (11.2)	52.5 (7.8)	8.1 (2.0)
**Sleep duration**				
<7 h	337 (33.0)	48.3 (17.6)	52.9 (10.7)	8.0 (2.0)
7–9 h	645 (63.1)	48.7 (17.3)	54.7 (9.7)	8.0 (2.0)
>9 h	40 (3.9)	42.0 (19.7)	50.9 (23.2)	7.0 (3.0)
**Sleep sufficiency, sleep duration**				
Low sufficiency, <7/>9 h	106 (10.4)	38.5 (18.3)	46.5 (22.2)	6.0 (3.0)
Low sufficiency, 7–9 h	89 (8.7)	34.2 (19.6)	54.1 (15.0)	6.0 (4.0)
High sufficiency, <7/>9 h	271 (26.5)	49.8 (15.5)	53.5 (9.6)	8.0 (2.0)
High sufficiency, 7-9 h	556 (54.4)	51.0 (15.1)	54.8 (9.1)	8.0 (2.0)

IQR, Interquartile Range.

**Table 3:** Adjusted associations (β coefficients, IC) between sleep sufficiency, sleep duration and health outcomes (mental health, physical health, and life satisfaction) (*n* = 1,022) (weighted data).

**Sleep variable**	**Mental health**		**Physical health**		**Life satisfaction**	
	β **(95% CI)**	***P-value***	β **(95% CI)**	***P-value***	β **(95% CI)**	***P-value***
**Sleep sufficiency**						
Low	−12.50 (−14.29; −10.71)	**<0.0001**	−3.05 (−4.47; −1.64)	**<0.0001**	−1.87 (−2.19; −1.55)	**<0.0001**
Moderate	−6.07 (−7.56; −4.59)		−2.00 (−3.15; −0.80)		−0.77 (−1.03; −0.51)	
High (ref.)	0		0		0	
**Sleep duration**						
<7 h	−0.73 (−2.27; 0.81)	**0.03**	−1.37 (−2.49; −0.25)	**0.02**	−0.34 (−0.60; −0.07)	**0.005**
7–9 h (ref.)	0		0		0	
>9 h	−4.45 (−7.89; −1.07)		−2.28 (−4.76; 0.21)		−0.73 (−1.32; −0.14)	

β, standardized regression coefficient; CI, Confidence Interval.

Negative β coefficients indicate lower score of health outcomes.

Statistically significant findings are highlighted in bold.

Adjusted for sex, age, chronic disease, and years of education.

All analyses were adjusted for sex, age, chronic disease, and years of education, based on a theory-driven approach and previous literature (Lallukka et al., 2018; Matsui et al., 2021; Seow et al., 2020). They were based on complete cases, including only participants with non-missing data for all variables used in the models.

In supplementary analyses, unadjusted analyses as well as logistic regression were performed to further explore the associations for the continuous outcomes. Logistic regression was included to examine results in a more interpretable form and to assess the robustness of the findings. Assumptions for linear and logistic regression were checked and met. Calibrated weights were constructed by Statistics Denmark and applied to all analyses to reduce the impact of non-response. These weights were based on information from administrative registers: sex, age, highest completed level of education, employment status, income, marital status, country of origin, number of visits to the general practitioner and hospitalizations, and home owner/tenant status (Hirshkowitz et al., 2015). All statistical procedures were conducted using SAS version 9.4.

## Results

Table 1 presents the characteristics of the study population. Women (63.4%), those aged 45 to 64 years (44.8%), those with a higher academic education (≥15 years) (54.9%) or absence of chronic diseases (57.7%) were the most likely to participate in the study.

Participants most commonly reported high sleep sufficiency (46.8%), a sleep duration of 7–9 h (63.1%; mean 7.3 h (SD ± 1.0), and a combination of both high sleep sufficiency and 7–9 h of sleep (54.4%) (Table 2). Individuals with either high sleep sufficiency or a sleep duration of 7–9 h had the highest median scores for mental health, physical health, and life satisfaction. In the joint categorization, individuals with a high sleep sufficiency who also slept 7–9 h also had the highest median scores across all three health outcomes: 51.0 for mental health, 54.8 for physical health, and 8.0 for life satisfaction.

Individuals with low sleep sufficiency had statistically significantly poorer mental health compared to those with high sleep sufficiency, with an average score 12.50 points lower (95% CI: −14.29; −10.71) (Table 3). Low sleep sufficiency was also associated with poorer physical health (−3.05 points, 95% CI: −4.47; −1.64) and lower life satisfaction (−1.87 points, 95% CI: −2.19; −1.55). Regarding sleep duration, individuals sleeping more than 9 h had the lowest scores across all three health outcomes, with the most pronounced association observed in mental health, showing a 4.45-point lower score (95% CI: −7.89; −1.07) compared to those sleeping 7–9 h. Unadjusted linear regression analyses (Supplementary Table S2) and adjusted logistic regression analyses (Supplementary Table S3) showed associations in the same direction and with similar statistical significance for mental health, physical health, and life satisfaction.

Figures 2 and 3 show that low sleep sufficiency combined with short (<7 h) and long (>9 h) sleep duration was significantly associated with lower scores in mental health, physical health, and life satisfaction, compared to those with high sleep sufficiency and sleeping 7–9 h, after adjusting for covariates. Additionally, even among those sleeping 7–9 h, low sleep sufficiency was significantly associated with lower scores in mental health and life satisfaction. The lowest score was observed among individuals with low sleep sufficiency and 7–9 h of sleep, with a 10.93 lower mental health score (95% CI: −13.24; −8.62) compared to those with the same amount of sleep but with high sleep sufficiency (Supplementary Table S4). Similarly, individuals with low sleep sufficiency sleeping <7 or >9 h had mental health scores 8.56 points lower (95% CI: −10.82; −6.25) than those with high sleep sufficiency in the same sleep duration category. Supplementary logistic regression analyses showed similar associations between the combined sleep outcomes and mental health, physical health, and life satisfaction (Supplementary Table S5).

## Discussion

Sleep is a fundamental determinant of health, but its multiple dimensions may affect health differently. The present study shows that while both short and long sleep durations were linked to poorer mental health, physical health, and life satisfaction, sleep sufficiency exhibited the strongest associations with these outcomes. Low sleep sufficiency was consistently associated with poorer health outcomes regardless of sleep duration, even in those with sleep durations adhering to sleep recommendations. This suggests that sleep sufficiency may be more strongly correlated to overall health than duration alone.

The findings of the present study align with previous studies examining sleep quality and sleep durations simultaneously. Seow et al. (2020) found that poor sleep, assessed using the Pittsburgh Sleep Quality Index (PSQI), was linked to mental and physical disorders regardless of self-reported sleep duration ( ≤ 6 h, 7–8 h, and ≥9 h) among individuals aged 18 years or older (Seow et al., 2020). Consistent findings were reported in a Japanese study, where sleep quality, also measured by the PSQI, demonstrated a more coherent association with mental and physical health than self-reported sleep duration ( ≤ 6 h, >6– ≤ 8 h, and >8 h) among individuals aged 20–69 years (Matsui et al., 2021). In a study from Australia, Lallukka et al. (2018) likewise found that poor sleep quality, defined by insomnia symptoms, was associated with worse physical, emotional and social functioning across different combinations of self-reported sleep durations (<6 h, 6–8 h, and >8 h) among individuals aged 15 or older (Lallukka et al., 2018). The strongest associations were observed for emotional and social functioning compared to physical functioning. This aligns with our findings, where associations were generally strongest for mental health and life satisfaction, supporting the notion that sleep quality plays an essential role in psychological wellbeing. Similarly, Seow et al. (2020) found that poor sleep in combination with <6 h of sleep was particularly linked to mental disorders such as major depressive disorder, bipolar disorder, and obsessive-compulsive disorder. These findings may indicate that sleep has a more immediate and noticeable impact on psychological wellbeing, which could be more sensitive to short-term sleep disturbances, whereas its impact on physical health may be more indirect, slower to manifest, or simply less pronounced.

Despite differences in how sleep quality (single item vs. multi-item instruments) and sleep duration (self-reported vs. device-based methods) were measured across studies, the overall patterns remain consistent, with sleep sufficiency emerging as a stronger predictor of health outcomes than sleep duration. While sleep duration is important, sleep sufficiency may better capture the restorative aspects of sleep, such as sleep efficiency, continuity, and depth (Kohyama, 2021). Individuals who get an adequate number of sleep hours may still experience sleep insufficiency due to frequent awakenings or difficulty falling asleep. In this sense, sleep duration is indirectly a component of sleep quality but does not fully determine whether sleep is restorative. Moreover, sleep recommendations are based on population averages (Hirshkowitz et al., 2015), but individual sleep needs vary, making subjective sleep sufficiency a more personalized and potentially more relevant measure.

### Strengths and limitations

A strength of the study was the use of the Danish Health and Morbidity Survey, a large, nationally representative survey of the general adult population, which included data on self-reported sleep sufficiency combined with objective, accelerometer-measured sleep duration (Jezek et al., 2024). Objective-measured sleep duration enhances the validity of the sleep duration estimates compared to studies relying on self-reports. Another strength of the study is that it is one of the few to have examined the joint effect of sleep duration and an indicator of sleep quality on different aspects of health. Further, it is the first study to use categories of sleep duration that align with the National Sleep Foundation's recommendations for adults and those used in many Western Countries, which is important as it enhances the applicability of the findings in public health and clinical practice. Additionally, by including both life satisfaction, reflecting overall life evaluation, and SF-12 outcomes, capturing health-related quality of life, the study provides complementary perspectives on wellbeing, encompassing both overall life evaluation and health-related functioning.

The study also has some limitations. Participants who completed the survey and wore an accelerometer represented a highly selected group, with a majority reporting no chronic diseases, high sleep sufficiency, adherence to the 7–9 h sleep recommendations, and high levels of mental and physical health. Participation in the accelerometer sub-study was relatively low (14.8% of those invited), which may introduce selection bias, particularly as only those who completed the full web-survey were invited to participate, making the sub-sample less representative of the general population from the outset. To address this, calibrated non-response weights were applied to the analysis.

An important methodological consideration relates the measurement of sleep. While sleep duration was assessed using a thigh-worn accelerometer, which is a validated method (Johansson et al., 2023), wrist-worn devices may capture certain aspects of sleep more directly. Our estimates of sleep duration also did not account for daytime napping, which could have provided a different picture of sleep duration. Moreover, the inclusion of additional sleep measures, such as device-measured sleep quality (efficiency, latency, depth, and continuity) and self-reported sleep duration, could have strengthened the study by providing a more comprehensive assessment of sleep dimensions.

Another limitation of the study is that, although the SF-12 (Gandek et al., 1998; Ware et al., 1996; Kontodimopoulos et al., 2007; Christensen et al., 2013), the Cantril ladder (OECD, 2013) and the use of accelerometers (Crowley et al., 2023) have all been validated, the single-item measure for sleep sufficiency has not. However, the simplicity of the single-item approach is a key strength in large-scale population-based surveys (Allen et al., 2022), and the consistency of our findings with those from studies using more comprehensive, validated instruments such as the PSQI suggests that even a simple measure on sleep sufficiency can reflect patterns in sleep-related health outcomes. Future studies should validate this measure against established sleep quality scales to ensure its reliability and accuracy.

Although the analyses were adjusted for sex, age, chronic disease, and years of education, residual confounding can never be completely ruled out. The measure of chronic disease may also be somewhat underestimated, as individuals who perceive their condition as well-managed or not currently limiting may not report having a long-standing illness. Furthermore, lifestyle factors such as physical activity, BMI, and smoking were not included in the models. This decision was based on the consideration that these variables may act as mediators rather than confounders in the relationship between sleep and health outcomes. Including them could therefore have led to overadjustment.

The cross-sectional design limits the ability to determine the directionality of associations between sleep duration, sleep sufficiency, and mental health, physical health, or life satisfaction and thus reverse causation is possible; individuals with poorer health may experience or perceive poorer sleep compared to those in better health. However, it is important to note that this study was not designed to assess causal relationships.

Further, longitudinal studies are needed to investigate the joint effects of sleep duration and sleep sufficiency on health outcomes. Even though the study population is large for an accelerometer-based study, the relatively small sample size in some of the groups (e.g., low sufficiency, 7–9 h) may affect the precision of the results, although the results are statistically significant. A larger population could also allow for more nuanced analyses of the joint effects by examining short (<7 h) and long (>9 h) sleep durations individually with sleep sufficiency in relation to health, rather than combining them. In the present study, results of the <7 h/9 h group primarily reflect individuals sleeping <7 h, as only *n* = 40 individuals reported sleeping >9 h. A larger sample would also enable moderation and subgroup analyses, exploring whether the associations differ by, for example sex and age.

In addition to refining both the methods and dimensions used to assess sleep, validating the single-item measure of sleep sufficiency, and including a larger sample size, future research should also explore potential mechanisms underlying the association between sleep sufficiency and health. These include behavioral and environmental factors related to sleep hygiene, such as screen use before bedtime, stress, rumination and poor sleep environment. A deeper understanding of these mechanisms could help inform interventions aimed at improving sleep health and preventing sleep-related health problems.

## Conclusion

Both self-reported low sleep sufficiency and accelerometer-measured sleep durations shorter and longer than the recommended 7–9 h are associated with poorer health outcomes, particularly mental health and life satisfaction. Notably, low sleep sufficiency had strong associations with health regardless of sleep duration. Thus, results of this study indicate that sleep sufficiency or sleep quality appears to be more strongly correlated to overall health than sleep duration alone, highlighting the need to integrate both dimensions into public health surveillance to better capture sleep-related health risks. Furthermore, the single-item measure of sleep sufficiency used in this study may be a feasible alternative to lengthy multi-item assessments of sleep quality in epidemiological studies, though validation studies are needed.

## Data Availability

The data analyzed in this study is subject to the following licenses/restrictions: The data that support the findings of this study are available on request from the corresponding author. The data are not publicly available due to privacy and ethical restrictions concerning participant confidentiality.

## References

[B1] Ahrensberg H. Møller S. R. Christensen A. I. Andersen S. Petersen C. B. (2025). Insufficient sleep in the Danish adult population: a 10-year trend analysis. Sleep Health. 11, 364–370. doi: 10.1016/j.sleh.2025.03.005 40268633

[B2] Allen M. S. Iliescu D. Greiff S. (2022). Single Item Measures in Psychological Science. Newburyport, MA: Hogrefe Publishing.

[B3] Alvarez G. G. Ayas N. T. (2004). The impact of daily sleep duration on health: a review of the literature. Progress Cardiovasc. Nurs. 19, 56–59. doi: 10.1111/j.0889-7204.2004.02422.x 15133379

[B4] Ancoli-Israel S. Cole R. Alessi C. Chambers M. Moorcroft W. Pollak C. P. (2003). The role of actigraphy in the study of sleep and circadian rhythms. Sleep 26, 342–392. doi: 10.1093/sleep/26.3.342 12749557

[B5] Buysse D. J. (2014). Sleep health: can we define it? Does it matter? Sleep. 37, 9–17. doi: 10.5665/sleep.3298 24470692 PMC3902880

[B6] Cantril H. (1965). The Pattern of Human Concerns. New Brunswic, NJ: Rutgers University Press.

[B7] Cappuccio F. P. Cooper D. D'Elia L. Strazzullo P. Miller M. A. (2011). Sleep duration predicts cardiovascular outcomes: a systematic review and meta-analysis of prospective studies. Eur. Heart J. 32, 1484–1492. doi: 10.1093/eurheartj/ehr007 21300732

[B8] Cappuccio F. P. D'Elia L. Strazzullo P. Miller M. A. (2010). Sleep duration and all-cause mortality: a systematic review and meta-analysis of prospective studies. Sleep. 33, 585–592. doi: 10.1093/sleep/33.5.585 20469800 PMC2864873

[B9] Chaput J. P. Dutil C. Featherstone R. Ross R. Giangregorio L. Saunders T. J. . (2020). Sleep duration and health in adults: an overview of systematic reviews. Appl. Physiol. Nutr. Metab. 45:S218–s31. doi: 10.1139/apnm-2020-0034 33054337

[B10] Chaput J. P. Wong S. L. Michaud I. (2017). Duration and quality of sleep among Canadians aged 18 to 79. Health Rep. 28, 28–33. Available online at: https://pubmed.ncbi.nlm.nih.gov/28930365/ 28930365

[B11] Chattu V. K. Manzar M. D. Kumary S. Burman D. Spence D. W. Pandi-Perumal S. R. (2018). The global problem of insufficient sleep and its serious public health implications. Healthcare 7:1. doi: 10.3390/healthcare7010001 30577441 PMC6473877

[B12] Christensen L. N. Ehlers L. Larsen F. B. Jensen M. B. (2013). Validation of the 12 item short form health survey in a sample from region Central Jutland. Soc. Indic. Res. 114, 513–521. doi: 10.1007/s11205-012-0159-9

[B13] Christensen A. I. Juel K. Kjøller M. Davidsen M. (2010). Mental sundhed blandt voksne danskere: analyser baseret på Sundheds-og sygelighedsundersøgelsen 2005. København: Sundhedsstyrelsen.

[B14] Clement-Carbonell V. Portilla-Tamarit I. Rubio-Aparicio M. Madrid-Valero J. J. (2021). Sleep quality, mental and physical health: a differential relationship. Int. J. Environ. Res. Public Health 18:460. doi: 10.3390/ijerph18020460 33435528 PMC7826982

[B15] Crowley P. Kildedal R. Vindelev S. O. Jacobsen S. S. Larsen J. R. Johansson P. J. . (2023). A novel system for the device-based measurement of physical activity, sedentary behavior, and sleep (Motus): usability evaluation. JMIR Form. Res. 7:e48209. doi: 10.2196/48209 37976096 PMC10692873

[B16] Damgaard A. J. Sørensen J. B. Jensen M. M. Pedersen P. (2024). The association between sleep, mental health, and health behaviours: a Danish population-based cross-sectional study. Scand. J. Public Health 11:14034948241262366. doi: 10.1177/14034948241262366 39129329

[B17] Frijters P. Krekel C. A. (2021). Handbook for Wellbeing Policy-Making: History, Theory, Measurement, Implementation, and Examples. Oxford: Oxford University Press.

[B18] Gandek B. Ware J. E. Aaronson N. K. Apolone G. Bjorner J. B. Brazier J. E. . (1998). Cross-validation of item selection and scoring for the SF-12 Health Survey in nine countries: results from the IQOLA Project. J. Clin. Epidemiol. 51, 1171–1178. doi: 10.1016/S0895-4356(98)00109-7 9817135

[B19] Goel N. Rao H. Durmer J. S. Dinges D. F. Eds. (2009). Neurocognitive Consequences of Sleep Deprivation. Seminars in Neurology. © New York: Thieme Medical Publishers. doi: 10.1055/s-0029-1237117 PMC3564638 19742409

[B20] Grandner M. A. (2019). “Epidemiology of insufficient sleep and poor sleep quality,” in Sleep and Health (Elsevier), 11–20. doi: 10.1016/B978-0-12-815373-4.00002-2

[B21] Hirshkowitz M. Whiton K. Albert S. M. Alessi C. Bruni O. DonCarlos L. . (2015). National Sleep Foundation's sleep time duration recommendations: methodology and results summary. Sleep Health 1, 40–43. doi: 10.1016/j.sleh.2014.12.010 29073412

[B22] Irwin M. R. (2015). Why sleep is important for health: a psychoneuroimmunology perspective. Ann. Rev. Psychol. 66, 143–72. doi: 10.1146/annurev-psych-010213-115205 25061767 PMC4961463

[B23] Jezek A. H. Ekholm O. Davidsen M. Petersen C. B. Rosendahl H. Møller S. R. . (2024). The Danish National Health Survey 2023: study design and participant characteristics. Scand. J. Public Health 8:14034948241275032. doi: 10.1177/14034948241275032 39380212

[B24] Johansson P. J. Crowley P. Axelsson J. Franklin K. Garde A. H. Hettiarachchi P. . (2023). Development and performance of a sleep estimation algorithm using a single accelerometer placed on the thigh: an evaluation against polysomnography. J. Sleep Res. 32:e13725. doi: 10.1111/jsr.13725 36167935 PMC10909528

[B25] Kohyama J. (2021). Which is more important for health: sleep quantity or sleep quality? Children 8:542. doi: 10.3390/children8070542 34202755 PMC8304732

[B26] Kontodimopoulos N. Pappa E. Niakas D. Tountas Y. (2007). Validity of SF-12 summary scores in a Greek general population. Health Qual. Life Outcomes 5, 1–9. doi: 10.1186/1477-7525-5-55 17900374 PMC2140054

[B27] Kronholm E. Partonen T. Härmä M. Hublin C. Lallukka T. Peltonen M. . (2016). Prevalence of insomnia-related symptoms continues to increase in the Finnish working-age population. J. Sleep Res. 25, 454–7. doi: 10.1111/jsr.12398 26868677

[B28] Lallukka T. Sivertsen B. Kronholm E. Bin Y. S. Øverland S. Glozier N. (2018). Association of sleep duration and sleep quality with the physical, social, and emotional functioning among Australian adults. Sleep Health. 4, 194–200. doi: 10.1016/j.sleh.2017.11.006 29555134

[B29] Lauderdale D. S. Knutson K. L. Yan L. L. Liu K. Rathouz P. J. (2008). Self-reported and measured sleep duration: how similar are they? Epidemiology 19, 838–45. doi: 10.1097/EDE.0b013e318187a7b0 18854708 PMC2785092

[B30] Li Y. Zhang X. Winkelman J. W. Redline S. Hu F. B. Stampfer M. . (2014). Association between insomnia symptoms and mortality: a prospective study of US men. Circulation 129, 737–746. doi: 10.1161/CIRCULATIONAHA.113.004500 24226807 PMC3987964

[B31] Liu Y. Croft J. B. Wheaton A. G. Perry G. S. Chapman D. P. Strine T. W. . (2013). Association between perceived insufficient sleep, frequent mental distress, obesity and chronic diseases among US adults, 2009 behavioral risk factor surveillance system. BMC Public Health 13:84. doi: 10.1186/1471-2458-13-84 23360346 PMC3562519

[B32] Liu Y. Wheaton A. G. Chapman D. P. Cunningham T. J. Lu H. Croft J. B. . (2016). Prevalence of healthy sleep duration among adults–United States, 2014. MMWR Morb. Mortal. Wkly. Rep. 65, 137–41. doi: 10.15585/mmwr.mm6506a1 26890214

[B33] Lucas R. E. Freedman V. A. Cornman J. C. (2018). The short-term stability of life satisfaction judgments. Emotion 18:1024. doi: 10.1037/emo0000357 28872337 PMC5835155

[B34] Martin J. L. Hakim A. D. (2011). Wrist actigraphy. Chest 139, 1514–1527. doi: 10.1378/chest.10-1872 21652563 PMC3109647

[B35] Matricciani L. Bin Y. S. Lallukka T. Kronholm E. Wake M. Paquet C. . (2018). Rethinking the sleep-health link. Sleep Health 4, 339–348. doi: 10.1016/j.sleh.2018.05.004 30031526

[B36] Matsui K. Yoshiike T. Nagao K. Utsumi T. Tsuru A. Otsuki R. . (2021). Association of subjective quality and quantity of sleep with quality of life among a general population. Int. J. Environ. Res. Public Health 18:12835. doi: 10.3390/ijerph182312835 34886562 PMC8657737

[B37] OECD (2013). OECD Guidelines on Measuring Subjective Well-being. Paris: OECD publishing. 24600748

[B38] Pedersen C. B. (2011). The Danish civil registration system. Scand. J. Public Health 39, 22–5. doi: 10.1177/1403494810387965 21775345

[B39] Pilcher J. J. Ginter D. R. Sadowsky B. (1997). Sleep quality versus sleep quantity: relationships between sleep and measures of health, well-being and sleepiness in college students. J. Psychosom. Res. 42, 583–596. doi: 10.1016/S0022-3999(97)00004-4 9226606

[B40] Rasmussen M. Santini Z. I. Joshanloo M. Petersen C. B. Christensen A. I. (2025). Determinants of life satisfaction and mental wellbeing in the danish general population: shared and distinct associations. Int. J. Public Health 70:1608531. doi: 10.3389/ijph.2025.1608531 41017793 PMC12463736

[B41] Ruggeri K. Garcia-Garzon E. Maguire Á. Matz S. Huppert F. A. (2020). Well-being is more than happiness and life satisfaction: a multidimensional analysis of 21 countries. Health Quality Life Outcomes 18, 1–16. doi: 10.1186/s12955-020-01423-y 32560725 PMC7304199

[B42] Scott A. J. Webb T. L. Martyn-St James M. Rowse G. Weich S. (2021). Improving sleep quality leads to better mental health: a meta-analysis of randomised controlled trials. Sleep Med. Rev. 60:101556. doi: 10.1016/j.smrv.2021.101556 34607184 PMC8651630

[B43] Seow L. S. E. Tan X. W. Chong S. A. Vaingankar J. A. Abdin E. Shafie S. . (2020). Independent and combined associations of sleep duration and sleep quality with common physical and mental disorders: results from a multi-ethnic population-based study. PLoS ONE 15:e0235816. doi: 10.1371/journal.pone.0235816 32673344 PMC7365445

[B44] Sofi F. Cesari F. Casini A. Macchi C. Abbate R. Gensini G. F. . (2014). Insomnia and risk of cardiovascular disease: a meta-analysis. Eur. J. Prev. Cardiol. 21, 57–64. doi: 10.1177/2047487312460020 22942213

[B45] Spytska L. (2024). The importance of quality sleep and its relationship with physical and mental health: a systematic review. Sleep Med. Res. 15, 162–72. doi: 10.17241/smr.2024.02264

[B46] Stamatakis E. Koster A. Hamer M. Rangul V. Lee I-. M. Bauman A. E. . (2020). Emerging collaborative research platforms for the next generation of physical activity, sleep and exercise medicine guidelines: the prospective physical activity, sitting, and sleep consortium (ProPASS). Br. J. Sports Med. 54, 435–437. doi: 10.1136/bjsports-2019-100786 31076396 PMC7146929

[B47] Stevens M. L. Gupta N. Eroglu E. I. Crowley P. J. Eroglu B. Bauman A. . (2020). Thigh-worn accelerometry for measuring movement and posture across the 24-hour cycle: a scoping review and expert statement. BMJ Open Sport Exercise Med. 6:e000874. doi: 10.1136/bmjsem-2020-000874 33408875 PMC7768971

[B48] Ware J. E. Kosinski M. Keller S. D. A. (1996). 12-Item Short-Form Health Survey: construction of scales and preliminary tests of reliability and validity. Med. Care. 34, 220–233. doi: 10.1097/00005650-199603000-00003 8628042

