# Supplementary material for: Comparison of self-reported sleep sufficiency and accelerometer-measured sleep duration in relation to mental health, physical health, and life satisfaction
Source: Front Sleep. 2025 Nov 26;4:1661250. doi: 10.3389/frsle.2025.1661250 (PMC12713883; doi:10.3389/frsle.2025.1661250)
Supplement: Supplementary file 1 [file Table_1.docx]

**Table S1.** Distribution of participants across sleep sufficiency and sleep duration categories (n=1,022). Numbers (%)

|  | **Sleep duration** |  |  |  |
| --- | --- | --- | --- | --- |
| **Sleep sufficiency** | <7 h | 7-9 h | >9 h | **Total** |
| Low | 92 (41.5) | 89 (49.1) | 14 (9.4) | 195 (100) |
| Moderate | 125 (35.8) | 217 (61.9) | 7 (2.3) | 349 (100) |
| High | 120 (25.4) | 339 (69.8) | 19 (4.8) | 478 (100) |
| **Total** | 337 (35.5) | 645 (62.6) | 40 (4.9) | 1,022 (100) |

**Table S2.** Unadjusted associations (β coefficients, CI) between sleep sufficiency, sleep duration and health outcomes (mental health, physical health and life satisfaction) (n=1,022).

|  | **Mental health** | | **Physical health** | | **Life satisfaction** | |
| --- | --- | --- | --- | --- | --- | --- |
|  | β (95% CI) | P-value | β (95% CI) | P-value | β (95% CI) | P-value |
| **Sleep sufficiency** | |  |  |  |  |  |
| Low | -14.06 (-15.85; -12.27) | **<.0001** | -4.07 (-5.66; -2.48) | **<.0001** | -2.18 (-2.49; -1.87) | **<.0001** |
| Moderate | -7.20 (-8.72; -5.69) |  | -1.74 (-3.90; -0.40) |  | -0.94 (-1.21; -0.68) |  |
| High (ref.) | 0 |  | 0 |  | 0 |  |
| **Sleep duration** | |  |  |  |  |  |
| <7 hours | 0.03 (-1.59; 1.64) | **0.01** | -1.97 (-3.27; -0.67) | **0.002** | -0.24 (-0.52; 0.03) | **0.002** |
| 7-9 hours (ref.) | 0 |  | 0 |  |  |  |
| >9 hours | -5.31(-8.81; -1.81) |  | -3.55 (-6.37; -0.74) |  | -0.99 (-1.59; -0.40) |  |

*β = standardized regression coefficient, CI = Confidence Interval.*

*Negative β coefficients indicate lower score of health outcomes.*

*Statistically significant findings are highlighted in bold.*

**Table S3.** Adjusted associations (OR 95 % CI) between sleep sufficiency, sleep duration and health outcomes (mental health, physical health and life satisfaction; high vs. low). Adjusted for sex, age, chronic disease and years of education (n=1,022).

|  | **Mental health^1^** | | **Physical health^1^** | | **Life satisfaction^2^** | |
| --- | --- | --- | --- | --- | --- | --- |
|  | OR (95% CI) | P-value | OR (95% CI) | P-value | OR (95% CI) | P-value |
| **Sleep sufficiency** | |  |  |  |  |  |
| Low | 0.10 (0.06; 0.17) | **<.0001** | 0.29 (0.15; 0.57) | **0.001** | 0.15 (0.09; 0.25) | **<.0001** |
| Moderate | 0.30 (0.19; 0.49) |  | 0.49 (0.26; 0.93) |  | 0.33 (0.24; 0.47) |  |
| High (ref.) | 1.00 |  | 1.00 |  | 1.00 |  |
| **Sleep duration** | |  |  |  |  |  |
| <7 hours | 0.87 (0.61; 1.25) | 0.08 | 0.71 (0.41; 1.22) | **0.02** | 0.64 (0.46; 0.88) | **0.02** |
| 7-9 hours (ref.) | 1.00 |  | 1.00 |  | 1.00 |  |
| >9 hours | 0.46 (0.23; 0.91) |  | 0.28 (0.11; 0.69) |  | 0.61 (0.28; 1.34) |  |

*OR = Odds Ratio, CI = Confidence Interval.*

*Negative β coefficients indicate lower score of health outcomes.
Statistically significant findings are highlighted in bold.*

*^1^Mental and physical health outcomes were dichotomized as high vs. low based on cut-off values from the Danish National Health Survey 2010. Individuals scoring below 35.76 (mental health) or below 35.37 (physical health) were categorized as having low health; these thresholds represent the lowest 10% of scores.
^2^Life satisfaction was dichotomized as high (score 9–10) vs. low (score <9), based on a median score (8.0) in the study population.*

**Table S4.** Associations (β coefficients, CI) between sleep sufficiency and sleep duration and health outcomes (mental health, physical health and life satisfaction) (n=1,022).

|  | **Model 1*** | | | | **Model 2**** | | | |
| --- | --- | --- | --- | --- | --- | --- | --- | --- |
|  | β | | 95 % CI | P-value | β | | 95 % CI | P-value |
| **Mental health** |  |  | |  |  |  | |  |
| Low sufficiency, <7/>9 h | -8.94 | (-11.29; -6.59) | | **<.0001** | -8.54 | (-10.83; -6.25) | | **<.0001** |
| Low sufficiency, 7-9 h | -12.84 | (15.22; -10.47) | |  | -10.93 | (-13.24; -8.62) | |  |
| High sufficiency, <7/>9 h | -0.33 | (-1.99; 1.32) | |  | -0.69 | (-2.29: 0.91) | |  |
| High sufficiency, 7-9 h (ref.) | 0 |  | |  | 0 |  | |  |
| **Physical health** |  |  | |  |  |  | |  |
| Low sufficiency, <7/>9 h | -6.67 | (-8.66; -4.68) | | **<.0001** | -4.37 | (-6.12; -2.62) | | **<.0001** |
| Low sufficiency, 7-9 h | -0.05 | (-2.07; 1.96) | |  | 0.07 | (-1.70; 1.83) | |  |
| High sufficiency, <7/>9 h | -0.40 | (-1.81; 1.00) | |  | -0.33 | (-1.55; 0.89) | |  |
| High sufficiency, 7-9 h (ref.) | 0 |  | |  | 0 |  | |  |
| **Life satisfaction** |  |  | |  |  |  | |  |
| Low sufficiency, <7/>9 h | -1.61 | (-2.02; -1.21) | | **<.0001** | -1.41 | (-1.81; -1.01) | |  |
| Low sufficiency, 7-9 h | -2.10 | (-2.50; -1.69) | |  | -1.82 | (-2.22; -1.42) | |  |
| High sufficiency, <7/>9 h | -0.32 | (-0.60; -0.03) | |  | -0.38 | (-0-66; -0.10) | | **<.0001** |
| High sufficiency, 7-9 h (ref.) | 0 |  | |  | 0 |  | |  |

*β = standardized regression coefficient, CI = Confidence Interval.*

*Negative β coefficients indicate lower score of health outcomes.*

*Statistically significant findings are highlighted in bold.*

**Unadjusted model.*

***Adjusted for sex, age, chronic disease and years of education.*

**Table S5.** Associations (OR 95 % CI) between sleep sufficiency and sleep duration and health outcomes (mental health, physical health and life satisfaction; high vs. low) (n=1,022).

|  | **Model 1*** | | **Model 2**** | |
| --- | --- | --- | --- | --- |
|  | OR (95 %CI) | p-value | OR (95 %CI) | p-value |
| **Mental health^1^** |  |  |  |  |
| Low sufficiency, <7/>9 h | 0.25 (0.15; 0.40) | **<.0001** | 0.24 (0.14; 0.40) | **<.0001** |
| Low sufficiency, 7-9 h | 0.13 (0.08; 0.22) |  | 0.16 (0.10; 0.26) |  |
| High sufficiency, <7/>9 h | 0.82 (0.54; 1.26) |  | 0.76 (0.49; 1.19) |  |
| High sufficiency, 7-9 h (ref.) | 1.00 |  | 1.00 |  |
| **Physical health^1^** |  |  |  |  |
| Low sufficiency, <7/>9 h | 0.21 (0.12; 0.38) | **<.0001** | 0.28 (0.15; 0.54) | **0.0005** |
| Low sufficiency, 7-9 h | 0.94 (0.40; 2.23) |  | 1.08 (0.42; 2.74) |  |
| High sufficiency, <7/>9 h | 1.10 (0.58; 2.08) |  | 1.12 (0.57; 2.20) |  |
| High sufficiency, 7-9 h (ref.) | 1.00 |  | 1.00 |  |
| **Life satisfaction^2^** |  |  |  |  |
| Low sufficiency, <7/>9 h | 0.21 (0.11; 0.40) | **<.0001** | 0.24 (0.12; 0.47) | **<.0001** |
| Low sufficiency, 7-9 h | 0.14 (0.07; 0.31) |  | 0.17 (0.08; 0.37) |  |
| High sufficiency, <7/>9 h | 0.70 (0.51; 0.97) |  | 0.66 (0.47; 0.93) |  |
| High sufficiency, 7-9 h (ref.) | 1.00 |  | 1.00 |  |

*OR = Odds Ratio, CI = Confidence Interval.
Statistically significant findings are highlighted in bold.*

**Unadjusted model.*

***Adjusted for sex, age, chronic disease and years of education.*

*^1^Mental and physical health outcomes were dichotomized as high vs. low based on cut-off values from the Danish National Health Survey 2010. Individuals scoring below 35.76 (mental health) or below 35.37 (physical health) were categorized as having low health; these thresholds represent the lowest 10% of scores.
^2^Life satisfaction was dichotomized as high (score 9–10) vs. low (score <9), based on a median score (8.0) in the study population.*
